# Eletrophilic Chemistry of Tranilast Is Involved in Its Anti-Colitic Activity via Nrf2-HO-1 Pathway Activation

**DOI:** 10.3390/ph14111092

**Published:** 2021-10-28

**Authors:** Seongkeun Jeong, Changyu Kang, Sohee Park, Sanghyun Ju, Jin-Wook Yoo, In-Soo Yoon, Hwayoung Yun, Yunjin Jung

**Affiliations:** College of Pharmacy, Pusan National University, Busan 46241, Korea; aofhd86@naver.com (S.J.); whale10000@naver.com (C.K.); psh7728@pusan.ac.kr (S.P.); jsh141002@naver.com (S.J.); jinwook@pusan.ac.kr (J.-W.Y.); insoo.yoon@pusan.ac.kr (I.-S.Y.); hyun@pusan.ac.kr (H.Y.)

**Keywords:** tranilast, electrophile, Nrf2, HO-1, colitis

## Abstract

Tranilast (TRL), a synthetic derivative of a tryptophan metabolite, is an anti-allergic drug used to treat bronchial asthma. We investigated how TRL activated the nuclear factor-erythroid 2 p45-related factor 2 (Nrf2)-hemeoxygenase-1 (HO-1) pathway based on the electrophilic chemistry of the drug and whether TRL activity contributed to the treatment of rat colitis. In human colon carcinoma cells, TRL activated Nrf2, as represented by an increase in nuclear Nrf2 and induction of Nrf2-dependent luciferase and, subsequently, HO-1, a target gene product of Nrf2. TRL activation of Nrf2 and induction of HO-1 were completely prevented by chemical reduction of the electrophilic functional group (α, β-unsaturated carbonyl group) in the drug. In parallel, TRL was reactive with the nucleophilic thiol group in *N*-acetylcysteine, forming a covalent adduct. Moreover, TRL, but not reduced TRL, binds to Kelch-like ECH-associated protein 1 (KEAP1), releasing Nrf2. TRL administration ameliorated colonic damage and inflammation in rats with dinitrobenzene sulfonic acid-induced colitis, which was partly compromised by the chemical reduction of TRL or co-treatment with an HO-1 inhibitor. Our results suggest that TRL activated the Nrf2-HO-1 pathway via covalent binding to KEAP1, partly contributing to TRL amelioration in rat colitis.

## 1. Introduction

Tranilast (TRL, *N*-[3′,4′-dimethoxycinnamoyl]-anthranilic acid) is a synthetic derivative of 3-hydroxyanthranilic acid, an immunomodulatory tryptophan metabolite [1]. TRL was initially developed and approved as an anti-allergic drug for the treatment of bronchial asthma. Later, TRL was also indicated for keloids and hypertrophic scars because TRL inhibits the proliferation of fibroblasts and suppresses collagen deposition in vivo. Recent studies have revealed the therapeutic potential of the drug for treating inflammatory diseases, including cancer, diabetes, and autoimmune, cardiovascular, and renal diseases [2].

Inflammatory bowel disease (IBD), representative of Crohn’s disease (CD) and ulcerative colitis (UC), is a chronic and incurable inflammation of the gastrointestinal (GI) tract with typical pathological manifestations, such as abdominal pain, diarrhea, and mucosal damage [3]. UC only affects the innermost lining of the colon, while CD can affect the entire GI tract and all bowel wall layers. The causes of both UC and CD are not known, and both diseases have similar contributing factors, such as environmental, genetic, and an inappropriate response by the body’s immune system [4,5]. In preclinical and clinical tests, TRL ameliorates experimental colitis and prevents intestinal stricture in CD patients [6,7,8,9,10], which is expected considering the aforementioned pharmacological activities of TRL.

Nuclear factor-erythroid 2 p45-related factor 2 (Nrf2), a cytoprotective transcription factor against oxidative stress, is sequestered in the cytoplasm by association with Kelch-like ECH-associated protein 1 (KEAP1), resulting in enhanced proteasomal Nrf2 degradation [11,12]. Under oxidative stress, Nrf2 is released from KEAP1 either by direct oxidative modification or after phosphorylation by redox-sensitive protein kinases. It then translocates to the nucleus, and in combination with other transcription factors, activates the gene transcription of a battery of antioxidative enzymes, resulting in a cytoprotective adaptive response [13,14,15]. These antioxidative enzymes attenuate inflammatory damage and neutralize the reactive oxygen species implicated in inflammatory signaling pathways [16].

Numerous in vivo studies, including experiments using Nrf2-deficient mice, have illustrated that Nrf2 plays an important role in modulating acute inflammation in various experimental models, including IBD. In fact, Nrf2-deficient mice exhibit increased susceptibility to dextran sulfate sodium-mediated colitis [17]. Moreover, pharmacological intervention with small-molecule Nrf2 signaling activators provides protection against experimental colitis [16,18,19].

Rectally or orally administered TRL is effective against various animal colitis models. The anti-colitic activity of TRL may be associated with induction of the anti-inflammatory enzyme hemeoxygenase-1 (HO-1) [8]. Since Nrf2 activation has a beneficial effect against colitis and HO-1 is a typical target gene of Nrf2 [16,20], we investigated whether TRL activated Nrf2, which contributed to the anti-colitic effect of TRL via HO-1 induction. We focused on the chemical structure of TRL, which possesses a potential electrophilic functional group (α, β-unsaturated carbonyl group, Michael acceptor) because an electrophile such as sulforaphane [21] is a well-known Nrf2 activator [22].

## 2. Results

### 2.1. TRL Activates the Nrf2-HO-1 Pathway in Human Colon Carcinoma Cells

**Figure 1:**
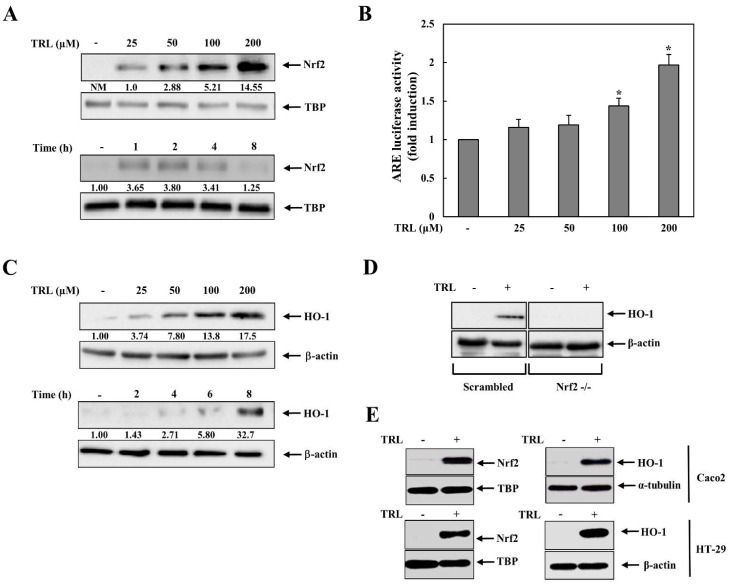
Tranilast (TRL) activates the Nrf2-HO-1 pathway. (**A**) Upper panel: HCT116 cells were treated with various concentrations of TRL for 2 h. The nuclear Nrf2 protein levels were assessed by western blotting. Lower panel: HCT116 cells were treated with TRL (200 μM) for the indicated times. The nuclear Nrf2 protein levels were assessed by western blotting. (**B**) Upper panel: HCT116 cells were treated with various concentrations of TRL for 8 h. The hemeoxygenase-1 (HO-1) protein levels were assessed by western blotting. Lower panel: HCT116 cells were treated with TRL (200 μM) for the indicated times. The HO-1 protein levels were assessed by western blotting. (**C**) HCT116 cells were cotransfected with an ARE-responsive luciferase reporter plasmid and a CMV *Renilla* luciferase plasmid for 24 h. The cells were treated with various concentrations of TRL for 8 h and ARE-responsive luciferase activity was measured and normalized to CMV *Renilla* luciferase activity. (**D)** Control (scrambled) and Nrf2-knockdown HCT116 cells (Nrf2-/-) were treated with TRL (200 μM) for 8 h. The HO-1 protein levels were assessed by western blotting. [E] Caco-2 cells and HT-29 cells were treated with TRL (200 μM). The nuclear Nrf2 and HO-1 protein levels were assessed after 2 and 8 h, respectively, by western blotting. For western blotting in (**A**,**C**,**D**,**E**), equivalent loading of proteins was verified using TATA box protein (TBP) for nuclear Nrf2 protein and β-actin for HO-1 protein. NM, not measurable. The data in (**B**) are presented as mean ± standard deviation (*n* = 3) *: *p* < 0.05 vs. control.

We examined whether TRL activated the Nrf2-HO-1 pathway in human colon carcinoma HCT116 cells. HCT116 cells were treated with various concentrations of TRL, and the levels of nuclear Nrf2 were monitored. As shown in Figure 1A (upper panel), nuclear Nrf2 increased in a dose-dependent manner. Temporal changes in nuclear Nrf2 levels were also examined. As shown in Figure 1A (lower panel), nuclear Nrf2 levels significantly increased after 1 h and returned to nearly baseline levels 8 h later. To verify whether the increase in nuclear Nrf2 was associated with its activation, a luciferase assay was conducted using an Nrf2-dependent luciferase plasmid. As shown in Figure 1B, TRL increased Nrf2-dependent luciferase activity. As a target gene product of Nrf2, HO-1 protein induction was tested in cells after treatment with TRL. As shown in Figure 1C (upper panel), as expected, TRL induced HO-1 protein expression, which was evident with 25 μM TRL, consistent with the result of nuclear Nrf2 accumulation (Figure 1A). Temporal changes in HO-1 levels are shown in Figure 3C (lower panel). HO-1 induction was observed at 4 h, and was evident at 8 h. Statistical results of Figure 1A,C are shown in Figure S1. To determine whether TRL induction of HO-1 was dependent on Nrf2, Nrf2-knockdown human colon carcinoma HCT116 cells (Nrf2-/-) were treated with TRL and HO-1 induction was examined. This experiment was repeated in control HCT116 cells stably transfected with scrambled shRNA (scHCT116). As shown in Figure 1D, TRL was able to induce HO-1 only in scHCT116 cells, indicating Nrf2-HO-1 pathway activation. The effect of TRL on the Nrf2-HO-1 pathway was also observed in other human colon carcinoma cell lines, HT-29 and Caco-2 cells (Figure 1E).

### 2.2. Reduced TRL Is Unable to Activate Nrf2

**Figure 2:**
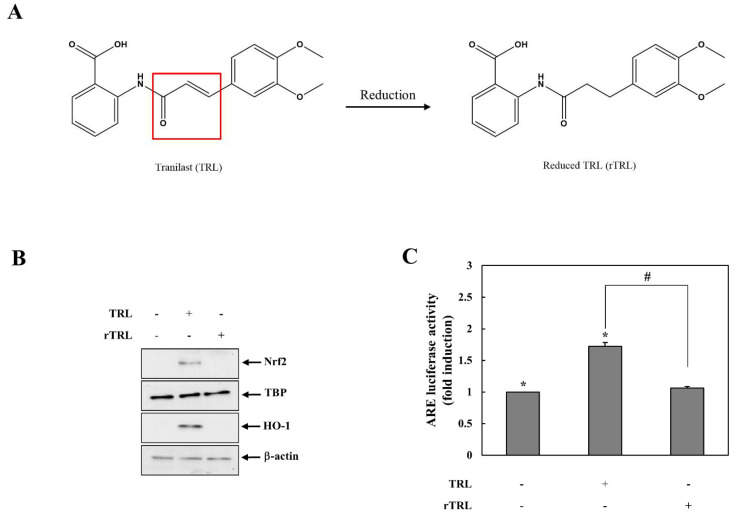
Reduced TRL is unable to activate Nrf2. (**A**) TRL was reduced to remove the electrophilic functional group in it. The box indicates the elctrophilic fuctional group. (**B**) HCT116 cells were treated with 200 μM of either TRL or rTRL. Nuclear Nrf2 and HO-1 protein levels were assessed after 2 and 8 h, respectively, by western blotting. (**C**) HCT116 cells were cotransfected with an ARE-responsive luciferase reporter plasmid and a CMV *Renilla* luciferase plasmid for 24 h. The cells were treated with 200 μM of either TRL or rTRL for 8 h. Luciferase activity was measured and normalized to CMV *Renilla* luciferase activity. For western blotting in (**B**), equivalent loading of proteins was verified using TATA box protein (TBP) for nuclear Nrf2 protein and β-actin for HO-1 protein. NM, not measurable. Data in (**C**) are presented as mean ± standard deviation (*n* = 3). *: *p* < 0.05 vs. control, #: *p* < 0.05.

We investigated the chemical mechanism by which TRL activates the Nrf2-HO-1 pathway. An electrophile is a typical Nrf2 activator [13], and TRL possesses an α, β-unsaturated carbonyl group, which is an electrophilic functional group [23]. Therefore, we hypothesized that TRL activates Nrf2 via the electrophilic chemistry. To test this hypothesis, TRL was chemically reduced to remove electrophilicity by saturation of the carbon-carbon double bond in the α,β-unsaturated carbonyl group as shown in Figure 2A. Activation of the Nrf2-HO-1 pathway by TRL was compared with that induced by reduced TRL (rTRL). However, as shown in Figure 2B,C in contrast to TRL, rTRL did not affect the levels of nuclear Nrf2, HO-1 induction, or Nrf2-dependent luciferase activity.

### 2.3. TRL Is Reactive with Thiol Group(s) in KEAP1 to Activate Nrf2

**Figure 3:**
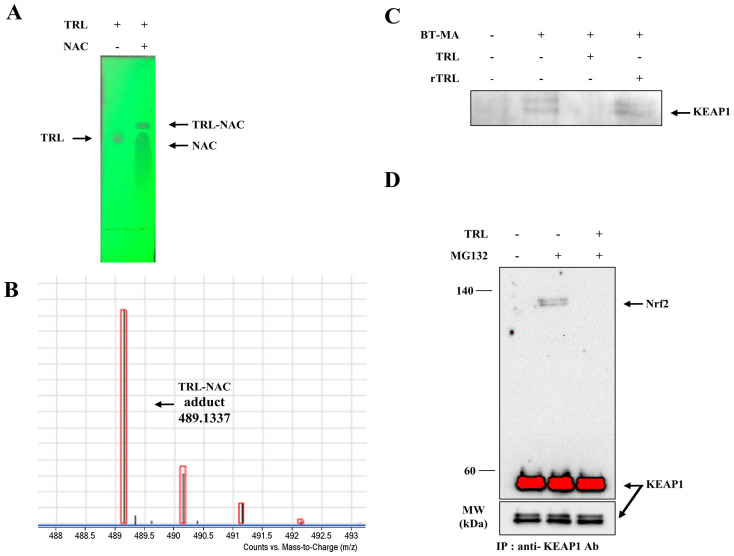
TRL reacts with a thiol group in KEAP1 to activate Nrf2. (**A**) TRL (200 μM) in acetonitrile/PBS (1/1, *v*/*v*) was incubated with or without 20 mM N-acetylcysteine (NAC) for 10 m. The reaction mixtures were analyzed by thin-layer chromatography. (**B**) The reaction mixture was subjected to mass spectrometry. The molecular weight peak (*m*/*z*, in negative mode) corresponding to the NAC-TRL adducts was detected. (**C**) Cell lysates were treated with 100 μM of either TRL or rTRL for 30 m, followed by incubation with a thiol-reactive biotin BT-MA (1 μM) at 30 °C for 2 h. The beads were washed five times. Laemmli sample buffer was added, and the mixture was boiled for 5 m prior to western blot analysis. The blots were probed with an anti-KEAP1 antibody. (**D**) HCT116 cells were pretreated with MG132 (10 μM) for 2 h and then lysed with lysis buffer. The cell lysates were treated with TRL (100 μM) at 30 °C for 2 h, followed by the addition of anti-KEAP1 antibody at 4 °C for 2 h. Protein A/G agarose beads were added to the lysates at 4 °C for 2 h. The beads were washed five times. Laemmli sample buffer was added, and the mixture was boiled for 5 min. Nrf2 and KEAP1 proteins in the immunocomplex were monitored by western blotting. NM, not measurable.

An electrophile binds KEAP1, a cytosolic repressor protein of Nrf2, to activate Nrf2 [22,24]. First, we examined whether TRL was reactive with a thiol group. TRL was incubated with N-acetylcysteine (NAC) with a nucleophilic thiol, and the reaction with the thiol was analyzed by TLC. As shown in Figure 3A, incubation of TRL with NAC changed the retardation factor (Rf) value of TRL, suggesting a reaction with NAC. When the same experiment was repeated with rTRL, no change was detected in the Rf value (data not shown). To identify an adduct produced from the reaction of TRL with NAC, a mass analysis was conducted to detect the covalent adduct. As shown in Figure 3B, the peak corresponding to the molecular weight of the TRL-NAC adduct was clearly detected at 489.1337. Expanded mass spectra of the TRL-NAC adduct and proposed structure of the adduct are shown in Figure S2. We next tested whether TRL binds to KEAP1 via thiol(s). Cell lysates were pretreated with TRL followed by the addition of biotin-maleimide (BT-MA), a thiol-reactive biotin, and proteins tagged with BT-MA were precipitated using streptavidin beads. KEAP1 in the precipitates was analyzed using western blotting. This experiment was repeated using rTRL. As shown in Figure 3C, BT-MA bound to KEAP1, causing precipitation by streptavidin beads. This was completely prevented by pretreatment with TRL. In contrast, rTRL did not affect the binding of BT-MA to KEAP1. This result indicates that TRL binds to KEAP1 via a reaction between the electrophilic functional group in TRL and the thiol groups in KEAP1. Moreover, we examined whether binding of TRL to KEAP1 led to the release of Nrf2 from the cytosolic repressor protein. Cell lysates were incubated with TRL, and Nrf2 was coimmunoprecipitated with a KEAP1 antibody, and Nrf2 was detected in the immunocomplex. As shown in Figure 3D, Nrf2 coimmunoprecipitated with KEAP1 in the cell lysates treated with the proteasome inhibitor MG132, and co-treatment with TRL completely prevented coimmunoprecipitation of Nrf2.

### 2.4. TRL Amelioration of Rat Colitis Is Partly Compromised by Either Chemical Reduction or an HO-1 Inhibitor

**Figure 4:**
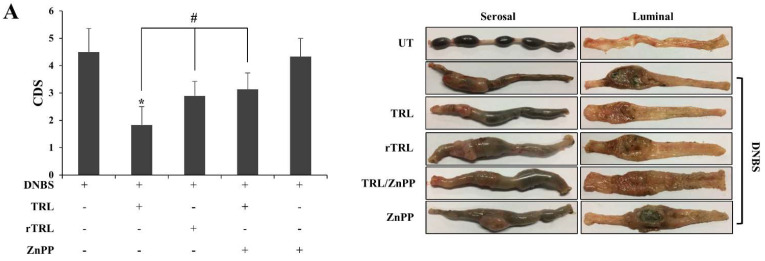

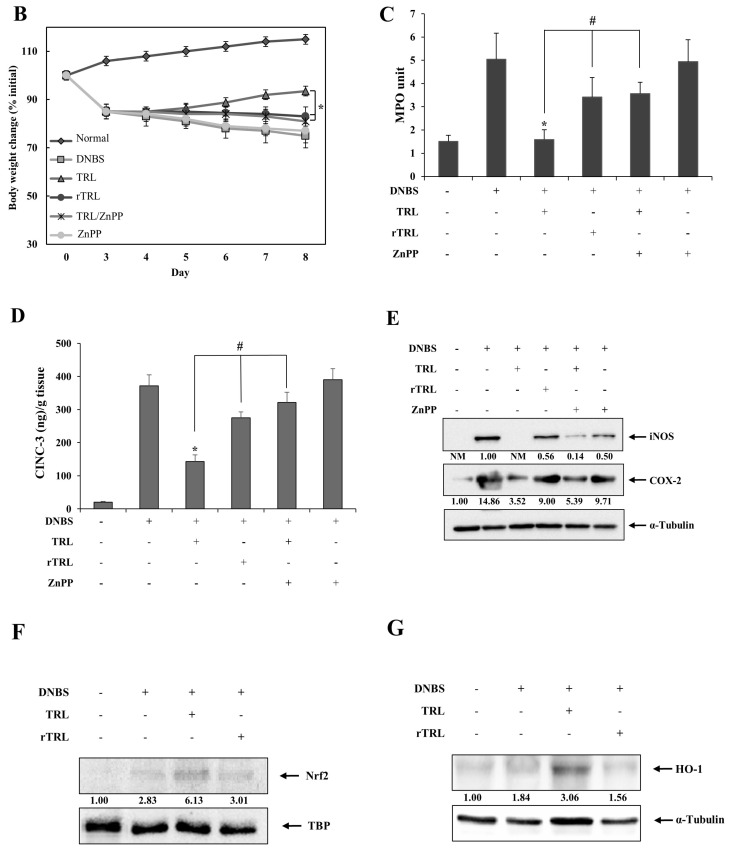
TRL amelioration of rat colitis is partly compromised by either its chemical reduction or co-treatment with an HO-1 inhibitor. Three days after colitis induction by DNBS, drugs were administered to rats once daily, and the rats were euthanized following the seventh treatment. The distal colons of rats were subjected to anti-colitic evaluation. (**A**) Left panel: The colonic damage score (CDS) was assessed using the modified scoring system. Right panel: The serosal and luminal sides of the distal colon were photographed. The images are representative of those obtained from the five rats. (**B**) Rat body weights were measured once a day and body weight change was presented as percent of initial weights. *: *p* < 0.05 (*n* = 5) (**C**) MPO activity was measured in the distal colon (4 cm). (**D**,**E**) The levels of inflammatory mediators such as CINC-3 (**D**), iNOS, and COX-2 (**E**) were monitored in the distal colon using a CINC-3 ELISA kit and western blotting. (**F**,**G**) Nuclear Nrf2 and HO-1 protein levels in the distal colon were monitored 2 h (for Nrf2) and 8 h (for HO-1) after rectal administration of either 200 μM of TRL or rTRL (in 500 μL PBS) by western blotting. For western blotting in (**E**–**G**), equivalent loading of proteins was verified using TATA box protein (TBP) for nuclear Nrf2 protein and α-tubulin for cytosolic proteins. Western blot results are representative of blots obtained from the distal colons of five rats. NM, not measurable. The data in (**A**–**C**) are expressed as the mean ± standard deviation (*n* = 5). *: *p* < 0.05 vs. control; #: *p* < 0.05.

TRL administered via the rectal or oral route is effective against experimental colitis models, and induction of HO-1 is known to be a pharmacological mechanism for the anti-colitic activities of TRL [8]. As our data indicate that TRL activates the Nrf2-HO-1 pathway via its electrophilic chemistry, we examined whether the chemistry of TRL is involved in the anti-colitic effects of TRL. TRL or rTRL was administered directly to the inflammatory distal colon of rats with dinitrobenzene sulfonic acid (DNBS)-induced colitis. The rectal route was used to minimize the influence of the pharmacokinetic factors. To further verify the involvement of HO-1 induction in TRL-mediated anti-colitic effects, the same experiment was performed with a mixture of TRL and zinc protoporphyrin IX (ZnPP), an HO-1 inhibitor. As shown in Figure 4A, DNBS induced severe colitis in the distal colon of rats, causing inflammatory damage, such as hemorrhagic ulcers, tissue edema, colonic obstructed stricture, and tissue adhesion with neighboring organs. TRL substantially ameliorated the inflammatory damage in the distal colon, and the ameliorative effect was partly impaired by the chemical reduction of TRL. The impairment in the ameliorative activity of TRL was similar to that of the combined HO-1 inhibitor treatment *(*Figure 4A; right panel). The colonic damage in each group was scored (Figure 4A; left panel). As shown in Figure 4B, the daily change in body weight was monitored as well. Body weight loss by the colitis was attenuated treatment with TRL, which showed significant difference with treatment with rTRL or co-treatment with TRL and ZnPP. MPO activity (representing neutrophil infiltration in colonic mucosa) elevated by colitis was decreased by TRL to a level close to that of the normal group, and chemical reduction of TRL or co-treatment with ZnPP partly undermined the ability of TRL to decrease MPO activity (Figure 4C). In addition, the levels of inflammatory mediators as molecular indices were examined in the distal colon. As shown in Figure 4D,E, inflammatory mediators (CINC-3, COX-2, and iNOS) were elevated in the inflamed colons. TRL also diminished CINC-3 levels, which were also partly undermined to a similar extent by chemical reduction or co-treatment with ZnPP. Regarding changes in iNOS and COX-2 levels, although chemical reduction of TRL undermined the TRL-mediated decrease in levels of the inflammatory mediators to a similar extent as shown in the results for CINC-3, impairment of the TRL effect by ZnPP was not as evident as that by ZnPP for CINC-3. Finally, we confirmed whether rectally administered TRL increased nuclear Nrf2 and HO-1 protein levels in the inflamed colon. This experiment was repeated using rTRL. As shown in Figure 4F,G, TRL, but not rTRL, increased the levels of proteins in the inflamed colon.

## 3. Discussion

In this study, we demonstrated that TRL activated the Nrf2-HO-1 pathway, and the Michael acceptor, an electrophilic functional group, was required in TRL for its molecular effect. Moreover, rectally administered TRL ameliorated colonic damage and inflammation in rats with DNBS-induced colitis, which was partly compromised by chemical reduction of TRL (removing electrophilicity) or co-treatment with an HO-1 inhibitor.

Consistent with previous papers [8,25], TRL induced HO-1 protein expression. Unlike one study demonstrating that TRL induction of HO-1 is dependent on the extracellular signal-regulated kinase-1/2 (ERK1/2) [25], our data indicate that TRL activation of Nrf2 results in HO-1 induction. This was verified in an experiment using Nrf2-knockdown HCT116 cells. In fact, TRL induction of HO-1 was prevented in Nrf2-knockdown HCT116 cells, while TRL clearly induced HO-1 in control HCT116 cells. Although we do not completely rule out the involvement of ERK1/2 in TRL-mediated Nrf2 activation, TRL activation of Nrf2 occurs largely by TRL binding to KEAP1. This argument is strongly supported by our data showing that TRL, which forms a covalent adduct by reacting with a thiol, effectively prevents thiol-reactive BT-MA from binding to KEAP1 and facilitates the release of Nrf2 from KEAP1. Moreover, the Michael acceptor in TRL is required for KEAP1 binding because, when electrophilicity in TRL is lost by chemical reduction, TRL actions, binding to KEAP1, and activation of Nrf2-HO-1 were completely abolished. Considering that a reactive electrophilic compound elicits various biological activities via reaction with functional proteins [26,27], it would be interesting to investigate whether the electrophilic chemistry of TRL plays a role in other biological effects of TRL involved in stabilizing mast cells, cell cycle and proliferation, apoptosis, angiogenesis, cell migration, and metastasis [2,28]. This investigation would also be valid for molecular targets of TRL, such as the TGF-β signaling pathway, mitogen-activated protein kinases (ERK, JNK, p38 MAPK), protein kinase C, NFκB, aryl hydrocarbon receptor, transient receptor potential channel vanilloid 2, S100 calcium-binding protein A11, and NLRP3 inflammasome [2,29,30].

Similar to previous studies [6,7,8], our study reported that TRL alleviated colonic damage and inflammation in experimental colitis. Rectally administered TRL healed colonic injury as estimated by CDS and improved inflammatory indicators in the inflamed colons, such as MPO activity and inflammatory mediators. The anti-colitic effects of TRL were ascribed, at least partly, to the activation of the anti-inflammatory Nrf2-HO-1 pathway. This argument is derived from our data showing that chemical reduction of TRL or co-treatment with an HO-1 inhibitor blunted the TRL effects against colitis. At the same time, our data clearly indicate that Nrf2-HO-1 pathway activation is a part of the anti-colitic pharmacological activity of TRL because rTRL, which has no ability to activate the Nrf2-HO-1 pathway, did not completely abolish the anti-colitic effects of TRL. TRL (200 μM) treated rectally increased Nrf2 and HO-1 protein levels in the inflamed colon, whereas rTRL at equimolar concentrations did not affect the protein levels, suggesting that the molecular effects can manifest in the colon or the rectum upon treatment of UC and proctitis with TRL enema. In addition, considering that oral TRL at the usual therapeutic dose (600 mg/day) affords plasma concentrations up to 300 μM [31], Nrf2-HO-1 pathway activation may contribute to the anti-colitic activity of TRL, regardless of the administration route.

The question now is what the other anti-colitic pharmacology of TRL is. Given that the molecular targets of TRL, such as NFκB, aryl hydrocarbon receptor, transient receptor potential channel vanilloid 2, and NLRP3 inflammasome, are potential drug targets for the development of an anti-colitic agent [32,33,34,35], it is possible to investigate the involvement of molecular targets in TRL-mediated anti-colitic effects, and the effects of rTRL on the molecular targets. Although chemical reduction of TRL and co-treatment with ZnPP similarly blunted anti-colitic effects (CDS, MPO activity, and CINC-3) of TRL, the compromised activity of rTRL may involve impaired binding to molecular targets because of loss of electrophilicity or structural change by chemical reduction. In fact, electrophiles affect the functions of some molecular targets, such as the NLRP3 inflammasome and NF-κB, via covalent binding [36,37]. In line with this, unlike the above-mentioned anti-colitic indicators, chemical reduction of TRL compromised the TRL-mediated decrease in COX-2 and iNOS levels more significantly than co-treatment with an HO-1 inhibitor, suggesting that there may be an additional pharmacology (other than Nrf2-HO-1 pathway) affected by chemical reduction of TRL.

In conclusion, TRL activates the Nrf2-HO-1 pathway via covalent binding to KEAP1, contributing to the amelioration of rat colitis as part of the anti-colitic activity of the drug. This study prompted us to revisit the molecular effects of TRL in view of the electrophilic chemistry of anti-allergic drugs.

## 4. Materials and Methods

### 4.1. Materials

Palladium on carbon (Pd/C, 10%) and BT-MA were purchased from Sigma Chemical Co. (St. Louis, MO, USA). TRL, DNBS, NAC, ZnPP, and NaBH4 were purchased from Tokyo Kasei Kogyo Co., Tokyo, Japan. The reaction solvents were obtained from Junsei Chemical Co. (Tokyo, Japan). All other chemicals were of reagent grade and were commercially available. The spots on thin-layer chromatography (TLC) plates (silica gel F254s, Merck Millipore, Burlington, MA, USA) were detected using a 254-nm ultraviolet lamp. Electrospray ionization mass spectrometry (ESI-MS) spectra were obtained using an Agilent 6530 Accurate-Mass Q-TOF LC/MS system (Agilent, Santa Clara, CA, USA). IR and 1H-NMR spectra were recorded using a Varian FT-IR spectrophotometer and a Varian AS 500 spectrometer (Varian, Palo Alto, CA, USA), respectively. The chemical shifts in the NMR spectra were reported in ppm downfield from tetramethylsilane.

### 4.2. Reduction of TRL

rTRL was obtained by chemical reduction of TRL in our laboratory as previously described [20]. rTRL synthesis was confirmed by TLC (methanol/chloroform [1:4]), FT-IR, 1H-NMR, and ESI-MS (in negative mode). The reaction scheme and structure of the rTRL are shown in Figure 2A. mp: 140 °C; IR (nujol mull), ν_max_ (cm^−1^): 1672 (C=O, carboxylic acid), 1604 (C=O, amide); 1H-NMR (DMSO-d6): δ = 8.48 (d, 1H), 7.96 (d, 1H), 7.58 (d, 1H), 7.14 (m, 1H), 6.87–6.82 (m, 2H), 6.75 (m, 1H), 3.71 (s, 3H), 3.69 (s, 3H), 2.88 (t, 2H), 2.69 (t, 2H); [M–H]-: *m*/*z* = 328.1046.

### 4.3. TRL-NAC Reaction

TRL (0.1 mM) dissolved in phosphate-buffered saline (PBS; pH 7.4) and acetonitrile (1:1, *v*/*v*, 1 mL) were treated with NAC (20 mM) at 30 °C for 10 min. The reaction mixture was then subjected to TLC (methanol/chloroform [1:4]) and ESI-MS (in negative mode).

### 4.4. Cell Culture and Transient Transfection

Human colon carcinoma HCT116, HT-29, and Caco-2 cells (ATCC, Manassas, VA, USA) were grown in Dulbecco’s modified Eagle’s medium (HyClone, Logan, UT, USA) supplemented with 10% fetal bovine serum (HyClone) and penicillin/streptomycin (HyClone). Nrf2-knockdown HCT116 cells and control cells stably expressing Nrf2 and non-specific scrambled shRNA, respectively, were established as previously described [38].

For transient transfection with an antioxidant responsive element (ARE)-responsive luciferase reporter gene plasmid (gifted from Prof. Kwak, The Catholic University of Korea, Republic of Korea), the cells were seeded in six-well plates, and grown to 50–60% confluence prior to transfection with the ARE-responsive luciferase plasmid (0.5 μg) and the cytomegalovirus (CMV) Renilla luciferase reporter plasmid (4 ng; Promega, Madison, WI, USA) using Fugene (Roche, Pleasanton, CA, USA) as a transfection reagent. After 24 h, the cells were treated for 6 h with TRL (25, 50, 100, and 200 μM) or rTRL (200 μM). The cells were lysed, and luciferase activity was measured and normalized to CMV Renilla luciferase activity using a Dual-Luciferase Reporter Assay Kit (Promega).

### 4.5. Immunoblot Analysis

After cell lysis, nuclear and cytosolic extracts were prepared as previously described [39]. To prepare distal colon tissue lysates, 0.2 g tissue samples were disrupted and homogenized in 2 mL of ice-cold radioimmunoprecipitation assay (RIPA) buffer (50 mM Tris-HCl (pH 7.4), 1 mM EDTA, 0.7% Na deoxycholate, 1% NP-40, 150 mM NaCl, 0.3 μM aprotinin, 1 μM pepstatin, and 1 mM phenylmethylsulfonyl fluoride (PMSF)). After incubation on ice for 30 min, the homogenates were centrifuged at 10,000× *g* at 4 °C for 10 min.

To prepare tissue nuclear extracts, 0.2-g distal colon samples were homogenized in 2 mL of ice-cold buffer C (10 mM HEPES (pH 7.9), 10 mM KCl, 0.2 mM EDTA, 0.3 mM aprotinin,1 mM pepstatin, and 1 mM PMSF). NP-40 (10%) was added to the homogenates at a final ratio of 64 μL/mL, followed by incubation for 20 min on ice. The mixture was vortexed vigorously for 15 s and centrifuged at 10,000× *g* at 4 °C for 5 min to obtain nuclear pellets. After removing the supernatants, an appropriate volume of buffer N (20 mM HEPES (pH 7.9), 0.4 M NaCl, 1 mM EDTA, 0.3 mM aprotinin, 1 mM pepstatin, and 1 mM PMSF) was added to the nuclear pellets and the tubes were rotated on a small rotary shaker at 4 °C for 60 min, and then centrifuged at 10,000× *g* at 4 °C for 10 min.

Protein concentrations in the supernatants obtained from the lysis process were determined using a bicinchoninic acid reagent according to the manufacturer’s instructions (Pierce, Rockford, IL, USA). The cell and tissue lysates were electrophoretically separated on 10% SDS-PAGE gels. Cyclooxygenase (COX)-2, inducible nitric oxide synthase (iNOS), KEAP1, and HO-1 proteins were detected using the anti-COX-2 (#4842, Cell Signaling Technology, Danvers, MA, USA), anti-iNOS (NOS-2) (sc-7271, Santa Cruz Biotechnology, Dallas, TX, USA), anti-KEAP1 (Santa Cruz Biotechnology), and anti-HO-1 (sc-390991, Santa Cruz Biotechnology) antibodies, respectively. Nrf2 protein was detected using an anti-Nrf2 antibody from Santa Cruz Biotechnology (sc-722). The bands were visualized using a SuperSignal chemiluminescence substrate (Pierce) in Chemidoc Touch Imaging System (BIO-RAD, Hercules, CA, USA). The experiments were normalized to TATA box-binding protein (TBP; Abcam, London, UK) or α-tubulin (Santa Cruz Biotechnology).

Western blot images were quantified using Image Lab software (version 5.2; build 14, Bio-Rad, Hercules, CA, USA). The quantified results were expressed as means from five animal and three cell experiments.

### 4.6. Precipitation of Biotin-Labeled Proteins and Coimmunoprecipitation

Cells were lysed using a lysis buffer (1% NP-40, 150 mM NaCl, 1 mM EDTA, 0.3 µM aprotinin, 1 µM pepstatin, and 1 mM PMSF). The cell lysates (0.7–0.8 mg protein) were incubated with BT-MA (1 μM) at 30 °C for 2 h in the presence or absence of 100 μM TRL or 200 μM rTRL. Streptavidin-agarose beads (20 µL; Millipore, Hayward, CA, USA) were added to the lysates and incubated at 4 °C for 2 h. The beads were washed five times with washing buffer (20 mM Tris pH 8, 100 mM NaCl, 1 mM EDTA, and 0.5% NP-40). For co-immunoprecipitation, cells pretreated with the proteasome inhibitor MG132 (Enzo Life Sciences, New York, NY, USA) for 2 h were lysed using RIPA buffer. The cell lysates were incubated at 30 °C for 2 h with TRL (100 μM), followed by the addition of the anti-KEAP1 antibody (sc-365626, Santa Cruz Biotechnology) at 4 °C for 2 h, after which protein A/G agarose (20 µL, Santa Cruz Biotechnology) was added at 4 °C for 2 h. The beads were washed five times with radioimmunoprecipitation assay (RIPA) buffer. SDS sample buffer (1×) was added, and the mixture was boiled for 5 m. The precipitated proteins were separated using 7.5% SDS-PAGE gels. Immunoblot analysis was performed with anti-Nrf2 antibody (Santa Cruz Biotechnology, sc-722) and anti-KEAP1 antibody as described above.

### 4.7. ELISA of CINC-3

To measure cytokine-induced neutrophil chemoattractant-3 (CINC-3) in inflamed tissues, the inflamed distal colon was homogenized in pH 6.0 sodium phosphate buffer, and centrifuged at 10,000× *g* at 4 °C for 10 min. An appropriate volume of the supernatant was subjected to a CINC-3 enzyme-linked immunosorbent assay (ELISA) kit (R&D Systems, Minneapolis, MN, USA).

### 4.8. Animals

Seven-week-old male Sprague-Dawley (SD) rats were purchased from Samtako Bio Korea (Kyeong-gi-do, South Korea) and housed in the animal care facility at Pusan National University, Busan, South Korea, in a facility with controlled temperature, humidity, and dark/light cycle. All animal experiments complied with the ARRIVE guidelines and were carried out in accordance with the National Institutes of Health Guide for the Care and Use of Laboratory Animals. The animal protocol used in this study was reviewed and approved by the Pusan National University–Institutional Animal Care and Use Committee (PNU–IACUC) for ethical procedures and scientific care (Approval No: PNU-2018-1898).

### 4.9. DNBS-Induced Rat Colitis

Experimental colitis was induced in rats as previously described [40]. Briefly, prior to colitis induction, male SD rats (250–260 g) were fasted for 24 h with free access to tap water. The rats were lightly anesthetized with isoflurane. A rubber cannula (2 mm OD) was inserted rectally such that the tip was 8 cm proximal to the anus, approximately at the splenic flexure. DNBS dissolved in 50% aqueous ethanol (*v*/*v*) was instilled into the colon via a rubber cannula (48 mg/0.4 mL/rat).

### 4.10. Evaluation of Anti-Colitic Effects

To evaluate the anti-colitic effects of TRL and the contribution of electrophilic chemistry to the TRL effects, six groups of five rats each were treated as follows: group 1 (UT), rectal administration of PBS (0.5 mL); group 2, rectal administration of PBS (0.5 mL); group 3, rectal administration of TRL (200 μM) in 0.5 mL PBS; group 4, rectal administration of rTRL (200 μM) in 0.5 mL PBS; group 5, rectal administration of TRL (200 μM) and ZnPP (20 μM) in 0.5 mL PBS; and group 6, rectal administration of ZnPP (20 μM) in 0.5 mL PBS.

Drugs dissolved (or suspended) in PBS were administered rectally to the rats once daily 3 days after the induction of colitis. After receiving medication for 7 days, the rats were euthanized using CO_2_ gas prior to evaluation of the anti-colitic effects. The colonic damage score (CDS) was calculated according to previously reported criteria [40]. The modified scoring system is shown in Figure S3. Four independent observers blinded to the treatment conditions performed CDS assessment. Myeloperoxidase (MPO) activity in the distal colon (4 cm) was measured as previously described [40].

### 4.11. Data Analysis

The results are expressed as mean ± standard deviation. One-way analysis of variance and either Tukey’s HSD test or the Mann-Whitney U test (for CDS) were used to test the difference between data. Statistical significance was set at *p* < 0.05. SigmaStat (SPSS Inc., Chicago, IL, USA) was used for the statistical analyses.

## 5. Conclusions

TRL activates the Nrf2-HO-1 pathway via covalent binding to KEAP1, contributing to the amelioration of rat colitis as part of the anti-colitic activity of the drug. This study prompted us to revisit the molecular effects of TRL in view of the electrophilic chemistry of anti-allergic drugs.

## Data Availability

Data is contained within the article or supplementary material.

## Supplementary Materials

The following are available online at https://www.mdpi.com/article/10.3390/ph14111092/s1, Figure S1. Statistical results of lower panel of Figure 1A (**A**), upper panel of Figure 1C (**B**), and lower panel of Figure 1C (**C**). The data are presented as mean ± standard deviation (*n* = 3) *: *p* < 0.05 vs. control. Figure S2. Structure (**A**) and expanded mass spectrum (**B**) of TRL-NAC adduct. Figure S3. Modified scoring system.
