# Supplementary material for: Eletrophilic Chemistry of Tranilast Is Involved in Its Anti-Colitic Activity via Nrf2-HO-1 Pathway Activation
Source: Pharmaceuticals (Basel). 2021 Oct 28;14(11):1092. doi: 10.3390/ph14111092 (PMC8623426; doi:10.3390/ph14111092)
Supplement: Supplementary file 1 [file pharmaceuticals-14-01092-s001.zip › pharmaceuticals-1405462-supplementary.pdf]

Figure S1

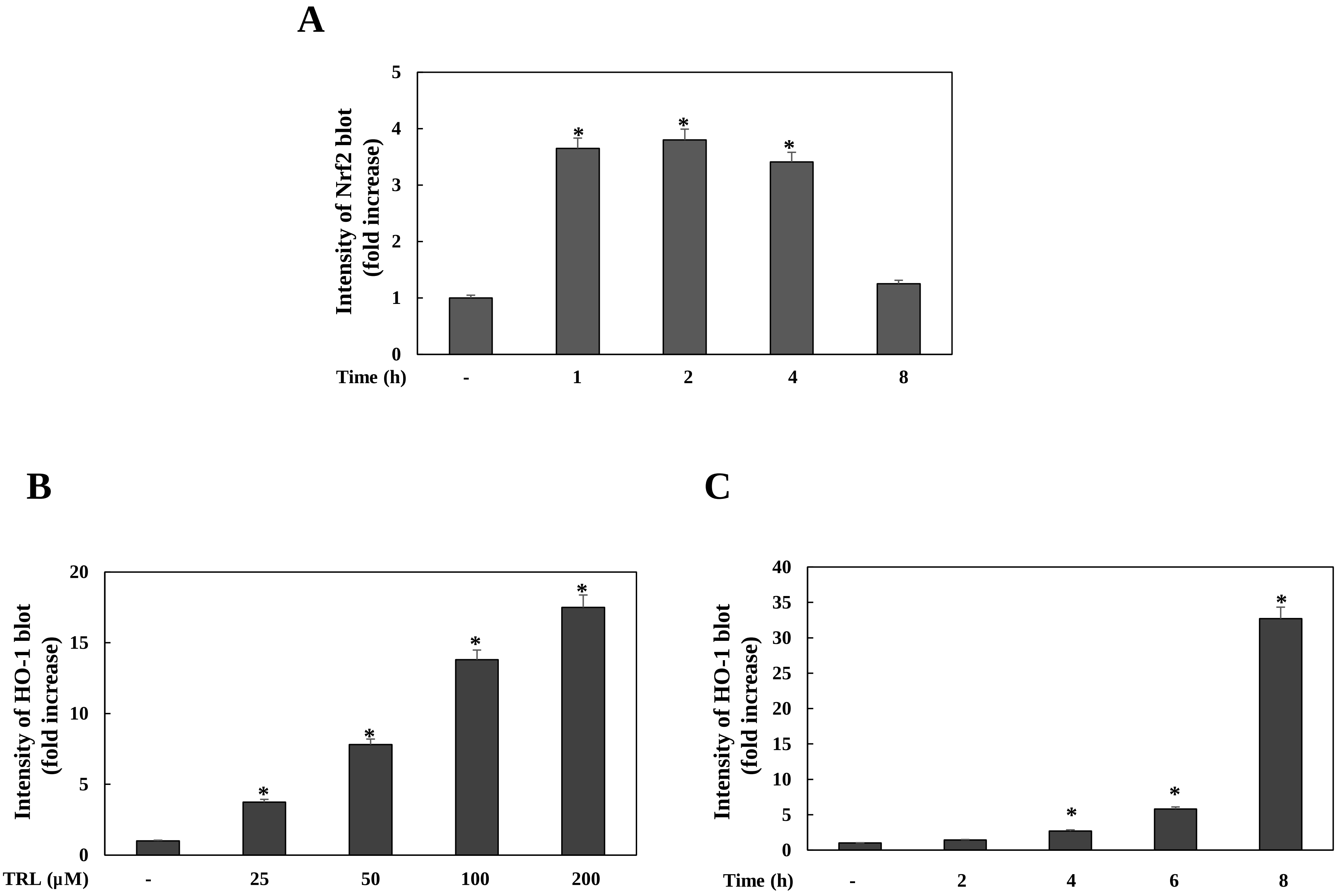

A
Intensity of Nrf2 blot (fold increase)
0
1
2
3
4
5
Time (h)
-
1
2
4
8
B
Intensity of HO-1 blot (fold increase)
0
5
10
15
20
TRL (μM)
-
25
50
100
200
C
Intensity of HO-1 blot (fold increase)
0
5
10
15
20
25
30
35
40
Time (h)
-
2
4
6
8

Figure S2

A

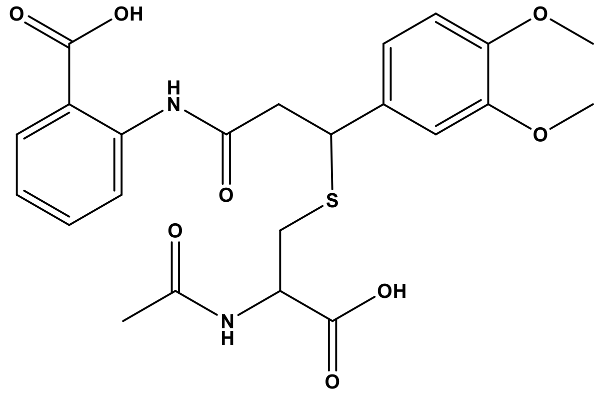

O
OH
H
N
O
O
O
S
O
N
H
OH
O

B

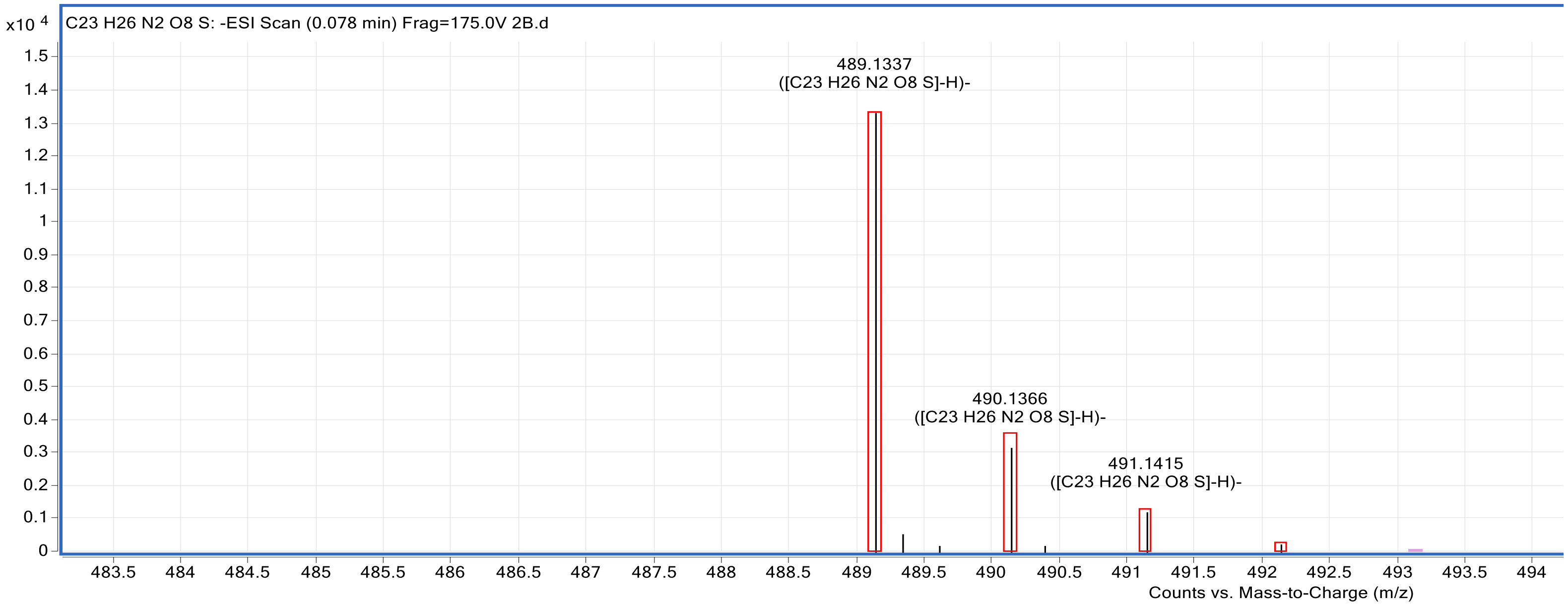

x10 4
C23 H26 N2 O8 S: -ESI Scan (0.078 min) Frag=175.0V 2B.d
1.5
1.4
1.3
1.2
1.1
1
0.9
0.8
0.7
0.6
0.5
0.4
0.3
0.2
0.1
0
489.1337
([C23 H26 N2 O8 S]-H)-
490.1366
([C23 H26 N2 O8 S]-H)-
491.1415
([C23 H26 N2 O8 S]-H)-
483.5
484
484.5
485
485.5
486
486.5
487
487.5
488
488.5
489
489.5
490
490.5
491
491.5
492
492.5
493
493.5
494
Counts vs. Mass-to-Charge (m/z)

Figure S3

Score	Feature
0	normal appearance
1	localized hyperemia but no ulcer
2	linear ulcers without significant inflammation
3	2–4 cm site of inflammation and ulceration
4	serosal adhesion to other organs, 2–4 cm site of inflammation and ulceration
5	stricture, serosal adhesion involving several bowel loops, <4 cm site of inflammation and ulceration
